# Tetra-μ-acetato-κ^8^ *O*:*O*′-bis­{[2,2-dimeth­yl-*N*-(pyridin-2-yl)propanamide-κ*N* ^1^]copper(II)}(*Cu*—*Cu*)

**DOI:** 10.1107/S1600536811050124

**Published:** 2011-11-30

**Authors:** Samuel Asem, Robert M. Buchanan, Mark S. Mashuta

**Affiliations:** a Department of Chemistry, University of Louisville, Louisville, KY 40292, USA

## Abstract

The crystal structure of the title compound, [Cu_2_(C_2_H_3_O_2_)_4_(C_10_H_14_N_2_O)_2_], reveals a dinuclear Cu^II^ complex located about a center of inversion. The coordination environment of each Cu^II^ cation is distorted octa­hedral, composed of four bridging acetate ligands, an apical pyridine donor and is completed by a Cu—Cu bond. The amide H atom forms intra­molecular hydrogen bonds to two carboxyl O atoms. In the crystal, weak inter­molecular pyridine–amide C—H⋯O inter­actions are also present.

## Related literature

For related paddlewheel structures, see: Aakeröy *et al.* (2003 ▶); Barquín *et al.* (2004 ▶, 2006 ▶); Fairuz *et al.* (2010 ▶); Seco *et al.* (2004 ▶); Sieroń (2004 ▶); Shi *et al.* (2008 ▶). For Cu⋯Cu separations in related compounds, see: Seco *et al.* (2004 ▶). For hydrogen bonding, see: Desiraju (1995 ▶). 
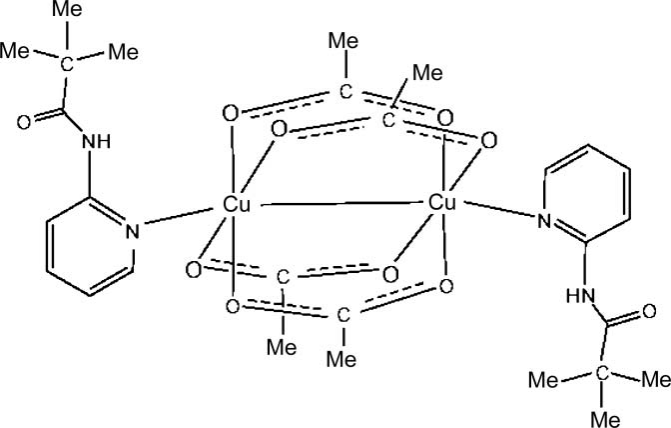


## Experimental

### 

#### Crystal data

[Cu_2_(C_2_H_3_O_2_)_4_(C_10_H_14_N_2_O)_2_]
*M* *_r_* = 719.74
Monoclinic, 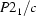

*a* = 13.8508 (8) Å
*b* = 11.0612 (5) Å
*c* = 11.0301 (6) Å
β = 104.508 (6)°
*V* = 1635.98 (15) Å^3^
*Z* = 2
Mo *K*α radiation
μ = 1.36 mm^−1^
*T* = 100 K
0.41 × 0.38 × 0.38 mm

#### Data collection

Oxford Diffraction Xcalibur Ruby Gemini diffractometer
Absorption correction: multi-scan (*CrysAlis PRO*; Oxford Diffraction, 2010 ▶) *T*_min_ = 0.581, *T*_max_ = 0.602
7567 measured reflections
3515 independent reflections
3070 reflections with *I* > 2σ(*I*)
*R*_int_ = 0.032

#### Refinement

*R*[*F* ^2^ > 2σ(*F* ^2^)] = 0.035
*wR*(*F* ^2^) = 0.094
*S* = 1.01
3515 reflections
208 parameters
H atoms treated by a mixture of independent and constrained refinement
Δρ_max_ = 0.73 e Å^−3^
Δρ_min_ = −0.58 e Å^−3^

### 

Data collection: *CrysAlis PRO* (Oxford Diffraction, 2009 ▶); cell refinement: *CrysAlis PRO* (Oxford Diffraction, 2010 ▶); data reduction: *CrysAlis PRO*; program(s) used to solve structure: *SHELXS97* (Sheldrick, 2008 ▶); program(s) used to refine structure: *SHELXL97* (Sheldrick, 2008 ▶); molecular graphics: *ORTEP-3* (Farrugia, 1997 ▶); software used to prepare material for publication: *SHELXTL* (Sheldrick, 2008 ▶), *PLATON* (Spek, 2009 ▶) and *publCIF* (Westrip, 2010 ▶).

## Supplementary Material

Crystal structure: contains datablock(s) global, I. DOI: 10.1107/S1600536811050124/sj5186sup1.cif

Structure factors: contains datablock(s) I. DOI: 10.1107/S1600536811050124/sj5186Isup2.hkl

Supplementary material file. DOI: 10.1107/S1600536811050124/sj5186Isup3.mol

Additional supplementary materials:  crystallographic information; 3D view; checkCIF report

## Figures and Tables

**Table 1:** Selected bond lengths (Å)

Cu1—O3	1.9670 (17)
Cu1—O2	1.9734 (17)
Cu1—O4	1.9739 (16)
Cu1—O1	1.9762 (16)
Cu1—N1	2.1990 (19)
Cu1—Cu1^i^	2.6168 (6)

Symmetry code: (i) 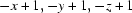.

**Table 2:** Hydrogen-bond geometry (Å, °)

*D*—H⋯*A*	*D*—H	H⋯*A*	*D*⋯*A*	*D*—H⋯*A*
N2—H2*N*⋯O4	0.80	2.38	3.118 (3)	154.1
N2—H2*N*⋯O2	0.80	2.71	3.226 (3)	124.0
C7—H7⋯O5^ii^	0.95	2.69	3.298 (3)	122
C8—H8⋯O5^ii^	0.95	2.63	3.262 (3)	124
C6—H6⋯O1^iii^	0.95	2.58	3.460 (3)	154

Symmetry codes: (ii) 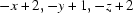; (iii) 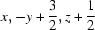.

**Fig. 1.**
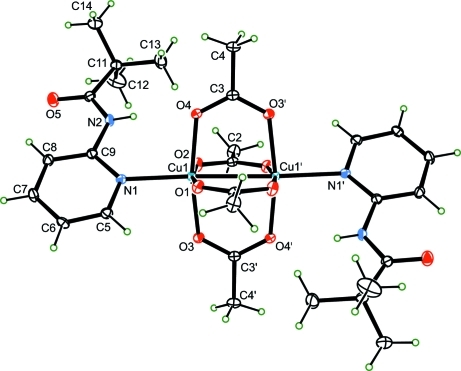
ORTEP-3 (Farrugia, 1997) view of (I) showing 50% displacement ellipsoids. H atoms are shown as small spheres of arbitrary radii.

**Fig. 2.**
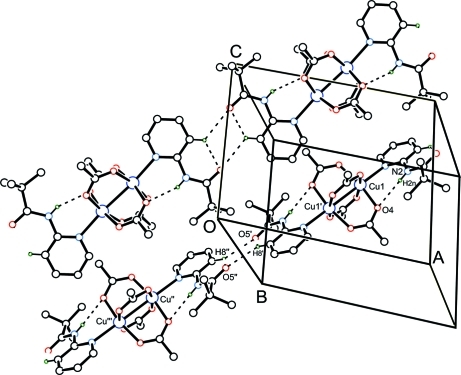
Packing diagram displaying hydrogen-bonding interactions. [Symmetry codes: (i) -x + 1, -y + 1, -z + 1 (ii) -x+2, 1 - y, -z] (iii) x, -y + 3/2, z + 1/2.

## supplementary crystallographic information

### Comment

Amide functionalized pyridine ligands have the potential to be used in the synthesis of supramolecular materials; particularly transition metal coordination polymers. The title complex, (I), is structurally similar to paddlewheel structures of other Cu_2_(OAc)_4_L_2_ complexes. (Aakeröy *et al.*, 2003; Barquín *et al.*, 2004, 2006; Fairuz *et al.*, 2010; Seco *et al.*; 2004; Sieroń, 2004; Shi, *et al.*, 2008). The dinuclear molecule lies about an inversion center.  Attached to this Cu_2_(OAc)_4_ core unit are two apical pyridine ligands functionalized in the 2-position of the ring with a pendant amide.  The average Cu-O (1.9726 (17) Å) and Cu-N (2.1990 (19) Å) distances and corresponding bond angles are consistent with structurally similar Cu^II^ complexes (Table 1).  While the Cu—Cu separation of 2.6168 (6) Å) is towards the lower limit, it is within the range of values reported for Cu^II^ paddlewheel structures. (Sieroń, 2004). The amide hydrogen forms intramolecular hydrogen bonds to two acetate oxygen atoms N2-H2- - -O4 and N2-H2- - -O2 (Table 2), (Desiraju, 1995). There are also three weak intermolecular C_py_-H- - -O amide interactions between adjacent metal complexes, C7-H7- - -O5, C8-H8- - -O5 and C6-H6- - -O1 (Table 2). These interactions result in the formation of infinite linear chains along the crystallographic AC diagonal and project through the BC face. The amide group and pyridine ring display a twist angle (C9-NH2-C10-O5) of 3.3 (4)°.

### Experimental

A stirred solution of (2-pivaloylamino)pyridine (0.4150 g, 2.328 mmol) in acetone (10 ml) was combined with a solution of Cu(CH_3_COO)_2_H_2_O (0.2324 g, 1.1642 mmol) in methanol (20 ml). The reaction was refluxed for an hour and stirring was continued for 24 h at room temperature. The reaction mixture was filtered and the solvent was removed *via* rotary evaporation. The resulting solid was dissolved in diethyl ether from which blue-green crystals deposited over several days.

### Refinement

The amide hydrogen atom was located from difference maps and refined isotropically. Aromatic H atom positions were calculated, and included as fixed contributions with *U*_iso_(H) = 1.2 *x U*_eq_(C). Methyl H atoms were placed in calculated positions and allowed to ride (the torsion angle which defines its orientation was allowed to refine) on the attached C atom, and these atoms were assigned *U*_iso_(H) = 1.5 *x U*_eq_(C). The highest peak, 0.73 e/Å^3^, and deepest trough, -0.58 e/Å^3^, are located 1.10 Å and 0.82 Å from Cu1 respectively.

### Crystal data

**Table:**

[Cu_2_(C_2_H_3_O_2_)_4_(C_10_H_14_N_2_O)_2_]	*F*(000) = 748
*M**_r_* = 719.74	*D*_x_ = 1.461 Mg m^−^^3^
Monoclinic, *P*2_1_/*c*	Mo *K*α radiation, λ = 0.71073 Å
Hall symbol: -P 2ybc	Cell parameters from 4128 reflections
*a* = 13.8508 (8) Å	θ = 3.6–28.8°
*b* = 11.0612 (5) Å	µ = 1.36 mm^−^^1^
*c* = 11.0301 (6) Å	*T* = 100 K
β = 104.508 (6)°	Block, blue-green
*V* = 1635.98 (15) Å^3^	0.41 × 0.38 × 0.38 mm
*Z* = 2	

### Data collection

**Table:**

Oxford Diffraction Xcalibur Ruby Gemini diffractometer	3515 independent reflections
Radiation source: Enhance (Mo) X-ray Source	3070 reflections with *I* > 2σ(*I*)
graphite	*R*_int_ = 0.032
Detector resolution: 10.2836 pixels mm^-1^	θ_max_ = 27.1°, θ_min_ = 3.6°
ω scans	*h* = −17→10
Absorption correction: multi-scan (*CrysAlis PRO*; Oxford Diffraction, 2010)	*k* = −11→13
*T*_min_ = 0.581, *T*_max_ = 0.602	*l* = −13→14
7567 measured reflections	

### Refinement

**Table:**

Refinement on *F*^2^	Primary atom site location: structure-invariant direct methods
Least-squares matrix: full	Secondary atom site location: difference Fourier map
*R*[*F*^2^ > 2σ(*F*^2^)] = 0.035	Hydrogen site location: inferred from neighbouring sites
*wR*(*F*^2^) = 0.094	H atoms treated by a mixture of independent and constrained refinement
*S* = 1.01	*w* = 1/[σ^2^(*F*_o_^2^) + (0.0434*P*)^2^ + 1.875*P*] where *P* = (*F*_o_^2^ + 2*F*_c_^2^)/3
3515 reflections	(Δ/σ)_max_ = 0.001
208 parameters	Δρ_max_ = 0.73 e Å^−^^3^
0 restraints	Δρ_min_ = −0.58 e Å^−^^3^

### Fractional atomic coordinates and isotropic or equivalent isotropic displacement parameters (Å^2^)

**Table:**

	*x*	*y*	*z*	*U*_iso_*/*U*_eq_	
Cu1	0.57135 (2)	0.51365 (2)	0.60219 (2)	0.01145 (10)	
O1	0.52507 (13)	0.68323 (15)	0.58335 (15)	0.0168 (4)	
O2	0.59958 (13)	0.33989 (15)	0.58943 (15)	0.0193 (4)	
O3	0.46567 (13)	0.47616 (16)	0.68677 (15)	0.0186 (4)	
O4	0.65766 (12)	0.54422 (16)	0.48765 (15)	0.0163 (4)	
O5	0.96072 (13)	0.38557 (17)	0.84694 (16)	0.0238 (4)	
N1	0.68384 (14)	0.55679 (18)	0.77605 (17)	0.0136 (4)	
N2	0.81361 (16)	0.4539 (2)	0.7259 (2)	0.0182 (4)	
H2N	0.778 (2)	0.455 (3)	0.657 (3)	0.017 (7)*	
C1	0.54803 (18)	0.2779 (2)	0.4997 (2)	0.0148 (5)	
C2	0.5762 (2)	0.1471 (2)	0.4927 (2)	0.0233 (6)	
H2A	0.5555	0.1020	0.5562	0.035*	
H2B	0.6472	0.1406	0.5056	0.035*	
H2C	0.5438	0.1153	0.4117	0.035*	
C3	0.62383 (18)	0.5437 (2)	0.3700 (2)	0.0144 (5)	
C4	0.6955 (2)	0.5688 (3)	0.2908 (2)	0.0229 (6)	
H4A	0.7117	0.4945	0.2555	0.034*	
H4B	0.7552	0.6044	0.3417	0.034*	
H4C	0.6653	0.6236	0.2247	0.034*	
C5	0.64649 (18)	0.6255 (2)	0.8544 (2)	0.0158 (5)	
H5	0.5800	0.6491	0.8289	0.019*	
C6	0.70168 (19)	0.6627 (2)	0.9702 (2)	0.0167 (5)	
H6	0.6730	0.7087	1.0225	0.020*	
C7	0.80085 (19)	0.6297 (2)	1.0061 (2)	0.0181 (5)	
H7	0.8404	0.6546	1.0832	0.022*	
C8	0.84148 (18)	0.5597 (2)	0.9276 (2)	0.0175 (5)	
H8	0.9082	0.5369	0.9508	0.021*	
C9	0.78030 (18)	0.5243 (2)	0.8134 (2)	0.0143 (5)	
C10	0.89929 (18)	0.3871 (2)	0.7467 (2)	0.0155 (5)	
C11	0.91516 (19)	0.3108 (2)	0.6368 (2)	0.0190 (5)	
C12	0.9151 (3)	0.1789 (3)	0.6784 (3)	0.0431 (9)	
H12A	0.8508	0.1587	0.6904	0.065*	
H12B	0.9649	0.1678	0.7556	0.065*	
H12C	0.9297	0.1274	0.6152	0.065*	
C13	0.8377 (2)	0.3303 (3)	0.5133 (3)	0.0352 (7)	
H13A	0.7731	0.3073	0.5225	0.053*	
H13B	0.8544	0.2818	0.4493	0.053*	
H13C	0.8368	0.4140	0.4902	0.053*	
C14	1.0181 (2)	0.3433 (3)	0.6186 (3)	0.0328 (7)	
H14A	1.0300	0.2981	0.5495	0.049*	
H14B	1.0683	0.3239	0.6933	0.049*	
H14C	1.0205	0.4282	0.6016	0.049*	

### Atomic displacement parameters (Å^2^)

**Table:**

	*U*^11^	*U*^22^	*U*^33^	*U*^12^	*U*^13^	*U*^23^
Cu1	0.01005 (16)	0.01279 (16)	0.01139 (16)	−0.00018 (11)	0.00250 (11)	−0.00009 (10)
O1	0.0154 (9)	0.0141 (8)	0.0198 (8)	0.0014 (7)	0.0025 (7)	−0.0011 (7)
O2	0.0190 (9)	0.0137 (8)	0.0222 (9)	0.0005 (7)	−0.0001 (7)	−0.0003 (7)
O3	0.0151 (9)	0.0253 (9)	0.0157 (8)	−0.0057 (7)	0.0046 (7)	0.0015 (7)
O4	0.0120 (8)	0.0230 (9)	0.0143 (8)	−0.0022 (7)	0.0040 (6)	0.0002 (7)
O5	0.0192 (9)	0.0297 (10)	0.0197 (9)	0.0091 (8)	−0.0006 (7)	−0.0054 (8)
N1	0.0120 (10)	0.0161 (10)	0.0133 (9)	0.0000 (8)	0.0042 (8)	−0.0010 (8)
N2	0.0124 (10)	0.0269 (12)	0.0137 (10)	0.0030 (9)	0.0003 (8)	−0.0074 (9)
C1	0.0143 (12)	0.0145 (11)	0.0170 (11)	0.0002 (9)	0.0067 (9)	0.0016 (9)
C2	0.0224 (14)	0.0155 (12)	0.0295 (14)	0.0029 (11)	0.0019 (11)	−0.0008 (10)
C3	0.0161 (12)	0.0112 (11)	0.0173 (11)	0.0011 (9)	0.0066 (9)	0.0006 (9)
C4	0.0197 (13)	0.0325 (15)	0.0190 (12)	−0.0029 (11)	0.0097 (10)	0.0022 (11)
C5	0.0142 (12)	0.0165 (12)	0.0169 (11)	0.0029 (9)	0.0045 (9)	0.0000 (9)
C6	0.0215 (13)	0.0159 (11)	0.0140 (11)	0.0031 (10)	0.0070 (10)	−0.0014 (9)
C7	0.0196 (13)	0.0189 (12)	0.0135 (11)	0.0011 (10)	−0.0002 (9)	−0.0034 (9)
C8	0.0132 (12)	0.0214 (12)	0.0166 (11)	0.0017 (10)	0.0016 (9)	−0.0021 (10)
C9	0.0147 (12)	0.0153 (11)	0.0133 (11)	0.0005 (9)	0.0040 (9)	−0.0007 (9)
C10	0.0131 (12)	0.0163 (12)	0.0177 (11)	−0.0012 (9)	0.0048 (9)	−0.0010 (9)
C11	0.0151 (12)	0.0231 (13)	0.0194 (12)	0.0038 (10)	0.0055 (10)	−0.0049 (10)
C12	0.073 (3)	0.0217 (15)	0.0434 (18)	−0.0007 (16)	0.0299 (18)	−0.0092 (14)
C13	0.0234 (15)	0.057 (2)	0.0236 (13)	0.0121 (14)	0.0026 (12)	−0.0191 (14)
C14	0.0220 (15)	0.0509 (19)	0.0284 (14)	0.0001 (14)	0.0116 (12)	−0.0073 (14)

### Geometric parameters (Å, °)

**Table:**

Cu1—O3	1.9670 (17)	C4—H4C	0.9600
Cu1—O2	1.9734 (17)	C5—C6	1.377 (3)
Cu1—O4	1.9739 (16)	C5—H5	0.9300
Cu1—O1	1.9762 (16)	C6—C7	1.380 (3)
Cu1—N1	2.1990 (19)	C6—H6	0.9300
Cu1—Cu1^i^	2.6168 (6)	C7—C8	1.382 (3)
O1—C1^i^	1.259 (3)	C7—H7	0.9300
O2—C1	1.267 (3)	C8—C9	1.387 (3)
O3—C3^i^	1.260 (3)	C8—H8	0.9300
O4—C3	1.264 (3)	C10—C11	1.537 (3)
O5—C10	1.215 (3)	C11—C13	1.524 (4)
N1—C9	1.345 (3)	C11—C12	1.529 (4)
N1—C5	1.348 (3)	C11—C14	1.532 (4)
N2—C10	1.368 (3)	C12—H12A	0.9600
N2—C9	1.405 (3)	C12—H12B	0.9600
N2—H2N	0.79 (3)	C12—H12C	0.9600
C1—C2	1.506 (3)	C13—H13A	0.9600
C2—H2A	0.9600	C13—H13B	0.9600
C2—H2B	0.9600	C13—H13C	0.9600
C2—H2C	0.9600	C14—H14A	0.9600
C3—C4	1.503 (3)	C14—H14B	0.9600
C4—H4A	0.9600	C14—H14C	0.9600
C4—H4B	0.9600		
			
O3—Cu1—O2	90.73 (8)	N1—C5—C6	123.4 (2)
O3—Cu1—O4	169.00 (7)	N1—C5—H5	118.3
O2—Cu1—O4	87.63 (8)	C6—C5—H5	118.3
O3—Cu1—O1	89.41 (7)	C5—C6—C7	118.0 (2)
O2—Cu1—O1	169.03 (7)	C5—C6—H6	121.0
O4—Cu1—O1	90.15 (7)	C7—C6—H6	121.0
O3—Cu1—N1	94.65 (7)	C6—C7—C8	120.0 (2)
O2—Cu1—N1	99.43 (7)	C6—C7—H7	120.0
O4—Cu1—N1	96.35 (7)	C8—C7—H7	120.0
O1—Cu1—N1	91.49 (7)	C7—C8—C9	118.4 (2)
O3—Cu1—Cu1^i^	83.86 (5)	C7—C8—H8	120.8
O2—Cu1—Cu1^i^	86.93 (5)	C9—C8—H8	120.8
O4—Cu1—Cu1^i^	85.19 (5)	N1—C9—C8	122.5 (2)
O1—Cu1—Cu1^i^	82.18 (5)	N1—C9—N2	114.2 (2)
N1—Cu1—Cu1^i^	173.50 (6)	C8—C9—N2	123.3 (2)
C1^i^—O1—Cu1	125.55 (15)	O5—C10—N2	122.7 (2)
C1—O2—Cu1	119.99 (15)	O5—C10—C11	120.3 (2)
C3^i^—O3—Cu1	123.85 (15)	N2—C10—C11	117.0 (2)
C3—O4—Cu1	121.86 (15)	C13—C11—C12	110.5 (3)
C9—N1—C5	117.8 (2)	C13—C11—C14	108.6 (2)
C9—N1—Cu1	129.93 (15)	C12—C11—C14	109.5 (3)
C5—N1—Cu1	112.31 (15)	C13—C11—C10	114.8 (2)
C10—N2—C9	127.2 (2)	C12—C11—C10	106.1 (2)
C10—N2—H2N	118 (2)	C14—C11—C10	107.2 (2)
C9—N2—H2N	115 (2)	C11—C12—H12A	109.5
O1^i^—C1—O2	125.2 (2)	C11—C12—H12B	109.5
O1^i^—C1—C2	117.5 (2)	H12A—C12—H12B	109.5
O2—C1—C2	117.2 (2)	C11—C12—H12C	109.5
C1—C2—H2A	109.5	H12A—C12—H12C	109.5
C1—C2—H2B	109.5	H12B—C12—H12C	109.5
H2A—C2—H2B	109.5	C11—C13—H13A	109.5
C1—C2—H2C	109.5	C11—C13—H13B	109.5
H2A—C2—H2C	109.5	H13A—C13—H13B	109.5
H2B—C2—H2C	109.5	C11—C13—H13C	109.5
O3^i^—C3—O4	125.2 (2)	H13A—C13—H13C	109.5
O3^i^—C3—C4	117.0 (2)	H13B—C13—H13C	109.5
O4—C3—C4	117.8 (2)	C11—C14—H14A	109.5
C3—C4—H4A	109.5	C11—C14—H14B	109.5
C3—C4—H4B	109.5	H14A—C14—H14B	109.5
H4A—C4—H4B	109.5	C11—C14—H14C	109.5
C3—C4—H4C	109.5	H14A—C14—H14C	109.5
H4A—C4—H4C	109.5	H14B—C14—H14C	109.5
H4B—C4—H4C	109.5		
			
O3—Cu1—O1—C1^i^	−80.42 (19)	O1—Cu1—N1—C5	45.82 (17)
O2—Cu1—O1—C1^i^	10.4 (5)	Cu1—O2—C1—O1^i^	−2.9 (3)
O4—Cu1—O1—C1^i^	88.59 (19)	Cu1—O2—C1—C2	177.59 (16)
N1—Cu1—O1—C1^i^	−175.05 (19)	Cu1—O4—C3—O3^i^	−0.3 (3)
Cu1^i^—Cu1—O1—C1^i^	3.46 (18)	Cu1—O4—C3—C4	179.58 (17)
O3—Cu1—O2—C1	84.00 (18)	C9—N1—C5—C6	−0.6 (4)
O4—Cu1—O2—C1	−85.12 (18)	Cu1—N1—C5—C6	178.83 (19)
O1—Cu1—O2—C1	−6.7 (5)	N1—C5—C6—C7	1.4 (4)
N1—Cu1—O2—C1	178.83 (17)	C5—C6—C7—C8	−1.0 (4)
Cu1^i^—Cu1—O2—C1	0.19 (17)	C6—C7—C8—C9	0.0 (4)
O2—Cu1—O3—C3^i^	−88.33 (19)	C5—N1—C9—C8	−0.6 (3)
O4—Cu1—O3—C3^i^	−7.0 (5)	Cu1—N1—C9—C8	−179.89 (18)
O1—Cu1—O3—C3^i^	80.70 (19)	C5—N1—C9—N2	−179.3 (2)
N1—Cu1—O3—C3^i^	172.15 (19)	Cu1—N1—C9—N2	1.3 (3)
Cu1^i^—Cu1—O3—C3^i^	−1.49 (18)	C7—C8—C9—N1	0.9 (4)
O3—Cu1—O4—C3	6.6 (5)	C7—C8—C9—N2	179.6 (2)
O2—Cu1—O4—C3	88.17 (18)	C10—N2—C9—N1	−165.0 (2)
O1—Cu1—O4—C3	−81.08 (18)	C10—N2—C9—C8	16.2 (4)
N1—Cu1—O4—C3	−172.60 (18)	C9—N2—C10—O5	−3.3 (4)
Cu1^i^—Cu1—O4—C3	1.05 (17)	C9—N2—C10—C11	175.9 (2)
O3—Cu1—N1—C9	135.7 (2)	O5—C10—C11—C13	−174.4 (3)
O2—Cu1—N1—C9	44.1 (2)	N2—C10—C11—C13	6.4 (3)
O4—Cu1—N1—C9	−44.5 (2)	O5—C10—C11—C12	63.3 (3)
O1—Cu1—N1—C9	−134.8 (2)	N2—C10—C11—C12	−115.9 (3)
O3—Cu1—N1—C5	−43.71 (17)	O5—C10—C11—C14	−53.6 (3)
O2—Cu1—N1—C5	−135.22 (16)	N2—C10—C11—C14	127.2 (2)
O4—Cu1—N1—C5	136.14 (16)		

Symmetry codes: (i) −*x*+1, −*y*+1, −*z*+1.

### Hydrogen-bond geometry (Å, °)

**Table:**

*D*—H···*A*	*D*—H	H···*A*	*D*···*A*	*D*—H···*A*
N2—H2N···O4	0.80	2.38	3.118 (3)	154.1
N2—H2N···O2	0.80	2.71	3.226 (3)	124.0
C7—H7···O5^ii^	0.95	2.69	3.298 (3)	122.
C8—H8···O5^ii^	0.95	2.63	3.262 (3)	124.
C6—H6···O1^iii^	0.95	2.58	3.460 (3)	154

Symmetry codes: (ii) −*x*+2, −*y*+1, −*z*+2; (iii) *x*, −*y*+3/2, *z*+1/2.
